# Aging changes the mechanism that underlies JAK2 modulation of neutrophil function

**DOI:** 10.1093/jimmun/vkaf323

**Published:** 2026-03-17

**Authors:** Jacob W. Feldmann, Matthew Kays, Farrah McGinnis, Emily Herron, Nurullah Sati, Clara Woods, Aminata P. Coulibaly

**Affiliations:** Department of Neuroscience, Rockefeller Neuroscience Institute, West Virginia University, Morgantown, WV, United States

**Keywords:** degranulation, JAK2, metabolism, migration, neutrophils

## Abstract

Janus kinase 2 (JAK2) has been linked to various neutrophil functions, but the intracellular mechanisms underlying its modulation are unknown. Neutrophils are essential cells for host defense. Neutrophil effector functions include migration, neutrophil extracellular trap production (NETosis), reactive oxygen species (ROS) production, and degranulation. The goal of this study was to elucidate the signaling mechanism through which JAK2 modulates neutrophil function and the effect of aging on this pathway. We hypothesized that JAK2-mediated modulation changes the molecular mechanisms associated with neutrophil function in an age-dependent manner. Neutrophils from young (3 mo) and aged (≥22 mo) male and female C57BL/6J mice were isolated, treated with a JAK2 inhibitor (AZD1480) or a pan-JAK inhibitor (baricitinib), and stimulated with PMA. Functional assays were conducted to assess migration, degranulation, NETosis, and metabolism. Mass spectrometry and Luminex assays provided proteomic and cytokine profiles. Our data showed that JAK2 promotes migration via membrane composition and actin remodeling, with age-dependent shifts in chemokine secretion. JAK2 primes ROS production by altering NADPH oxidase components, which contributes to NET production. JAK2 influences degranulation through actin remodeling. While aged neutrophils display impaired ROS-granule release, both young and aged neutrophils have distinct JAK-dependent release of granule contents. Metabolically, JAK2 enhances pentose phosphate pathway activity in young neutrophils and decreases glycogen breakdown in aged cells. These findings reveal mechanisms by which JAK2 modulates neutrophil function and suggest that organismal age plays a role in this modulation.

## Introduction

Neutrophils are the most abundant innate immune cell in the mammalian body. They are the first immune cells recruited to sites of inflammation, injury, and infection.^1–3^ Neutrophils comprise 40% to 80% of all white blood cells in the mammalian system.^4^ Neutrophils have short half-lives, leading to a high turnover rate. Due to their importance in the inflammatory response and abundance in circulation, it is important to fully understand the biology of this immune cell type. Neutrophil effector functions are critical to their activity. These effector functions include production of cytokines to mediate pro- or anti-inflammatory environments, removal of debris and damaged cells through phagocytosis, production of reactive oxygen species (ROS) in order to aid in cellular breakdown or cell signaling,^5^ and release of neutrophil extracellular traps (NETs).^6^ These functions are modulated by various intracellular signaling cascades initiated by various ligands and receptor interactions at the cell plasma membrane. It is evident that various physiological factors affect neutrophil function, including aging. A study using human bone marrow from young (20 to 30 years of age) and aged (70 to 80 years of age) healthy volunteers showed blunted response to granulocyte colony-stimulating factor (G-CSF) with age.^7^ In mice, circulating neutrophil numbers increase with age (with male neutrophils increasing at a faster rate compared to females), in a nondisease state.^8^ These data suggest that age directly affects neutrophil response to stimuli, but whether this is due to a change in intracellular integration of signals is unknown.

The JAK/STAT pathway is a ubiquitous signaling mechanism across various cell types, activated by extracellular stimuli like inflammatory cues (cytokines/chemokines) and growth factors.^9–14^ There are 4 characterized JAK isoforms: JAK1, JAK2, JAK3, and TYK2. The activation of each JAK protein elicits different downstream effector proteins, which lead to different functional outcomes.^15–18^ Our current understanding of JAK/STAT signaling in neutrophils is limited. JAK2 activation was shown to activate neutrophils, regulate migration,^19^ increase proliferation of neutrophil progenitor cells,^20,21^ and enhance neutrophil fungal killing through the release of elastase, matrix metalloprotease 9 (MMP-9), and ROS.^22^ In this project we aimed to elucidate whether age affects JAK modulation of neutrophil function. We hypothesized that age changes the intracellular pathway used by JAK2 to modulate neutrophil activity.

Our data showed that aging abrogates the direct effect JAK2 activation has on neutrophil function. JAK2 activation in neutrophils from young mice inhibited the release of stromal cell-derived factor (SDF)-1α and increased nucleation of actin to promote migration. In neutrophils from aged mice, JAK2 activation increased the release of CXCL2 and increased actin nucleation, suggesting an autocrine regulation of neutrophil migration. JAK2 activation suppressed secondary and tertiary granule release and increased secretory vesicle release in neutrophils from both young and aged mice. JAK2 signaling played a critical role in glucose metabolism in young and aged neutrophils. JAK2 facilitated the activation of the pentose phosphate pathway (PPP) in neutrophils from young mice, while regulating glycogen breakdown only in neutrophils from aged mice. This study provides evidence of various cellular mechanisms in neutrophils that are modulated by JAK2 activation in an age-dependent manner.

## Materials and methods

### Animals

Young (3 mo of age) and aged (≥22mo of age) wild-type (C57Bl6/J) mice were generated in our animal facility or purchased from The Jackson Laboratory. Functional assays were conducted using 4 to 6 mice per sex/age group. Each mouse provided a sufficient number of cells to be allocated across all experimental conditions, enabling their use in functional assays and mass spectrometry analysis. Mice were housed under normal conditions, with a 12:12h light:dark cycle, and had access to food and water ad libidum. All animal procedures were approved by the Institutional Animal Care and Use Committee at West Virginia University (WVU).

### Neutrophil isolation

Mice were euthanized by isoflurane overdose, and bone marrow was collected using sterile RPMI solution with 2mM EDTA. Neutrophils were isolated from the cell suspension using the negative selection MojoSort Kit (BioLegend, catalog #480058). Protease and phosphatase inhibitor cocktail (Thermo Fisher, catalog #78442) was added during the isolation process to minimize protein degradation. This isolation yields on average 3.8×10^6^ cells/mL in 4mL of media. Flow analysis shows an 80% neutrophil enrichment post-isolation, compared to the pre-isolation of 40% neutrophils (Fig. S1A).

### Culture preparation and collection for functional and proteomic analysis

For functional assays using immunohistochemistry (IHC), isolated neutrophils were plated on a poly-d-lysine–coated glass-bottom petri dish with RPMI media at a density of 2.5×10^5^ cells/mL. For the migration assay, glycolysis assay, and proteomic collection, plates were not treated with poly-d-lysine. Cultures were placed in the cell culture incubator (at 37 °C with 95% humidity and 5% CO_2_) for 30 min. Inhibitors (1 μM; baricitinib, Selleckchem, catalog #S2851; or AZD1480, AdooQ, catalog #A10110) were added to cultures for 1h, followed by the addition of phorbol 12-myristate 13-acetate (PMA) (32.43 nM/20 ng/mL; Sigma-Aldrich, catalog #524400) for an additional 45min, prior to collection (except for the migration assay). For proteomic collection, cultures were transferred to FACS tubes and spun at 300×*g* for 10min, supernatants and cell pellets were collected, and a protease and phosphatase inhibitor cocktail was added. These samples were flash frozen and stored until sent to IDeA National Resource for Quantitative Proteomics core at the University of Arkansas for Medical Sciences for mass spectrometry.

### Functional assays

#### Migration assay

Boyden chamber insets, with 3-μm pores, were purchased from Abcam (catalog #ab235692). These transwell chambers were placed on a 96-well plate; the lower chamber of the well contained 150μL of media and the chemoattractant IL-8 (20 ng/mL; Kingfisher BioTech, catalog #RP0357H-005). A total of 5×10^4^ neutrophils was added to the top chamber of each transwell in 100μL, and PMA (20ng/mL) or G-CSF (1ng/mL) was added to each well. Cultures were then left to incubate for 2h in the incubator. At the end of the incubation period, the number of cells in the lower chamber was counted using the Countess 3 automated cell counter (Fisher Scientific).

#### Modified CellROX assay

Isolated neutrophils were plated on a poly-d-lysine–coated glass-bottom petri dish. Then, 2.5×10^5^ neutrophils were added for the ROS degranulation assays. CellROX green reagent (5μM; Thermo Fisher, catalog #C10444) was applied to the cells at the same time as the PMA application (after the inhibitor incubation as stated above). After 45min, plates were collected, aspirated, and fixed with 4% paraformaldehyde (Electron Microscopy Sciences, catalog #30525-89-4) for 1h. Cells were counterstained with DAPI (Thermo Scientific, catalog #62248) and phalloidin (Thermo Fisher, catalog #A22287). Plates were blinded for image analysis and quantification. Three images were obtained per plate using the epifluorescent Echo Revolve. For quantification purposes, cells that had green fluorescence immediately adjacent to the phalloidin cell boundary were counted as degranulated cells. Data were normalized by the total number of DAPI nuclei in each image prior to analysis.

### Mass spectrometry

Frozen supernatants (secretome) and cell pellet (cell fraction) samples (*n*=4/sex/group) were sent to the IDeA National Resource for Quantitative Proteomics core for processing and analysis. The acquired proteomics data were post-processed and grouped based on condition and age. Data normalization was performed using a Loess smoothing model, which employs locally weighted regression to estimate and correct for bias. Contrast comparison values, including log fold changes, false discovery rate–adjusted *P* values, and average intensities, were normalized to control and obtained from IDeA. Subsequently, in-house analyses were conducted using Ingenuity pathway analysis (Qiagen) to examine differences across conditions and age groups. Proteins that fell below the detection threshold in some samples were excluded from the entire group to maintain a consistent sample size of at least *n*=3, ensuring statistical comparison. All comparisons were normalized to age- and sex-matched controls.

### Venn diagram and Gene Ontology term analysis

**Figure 1.**
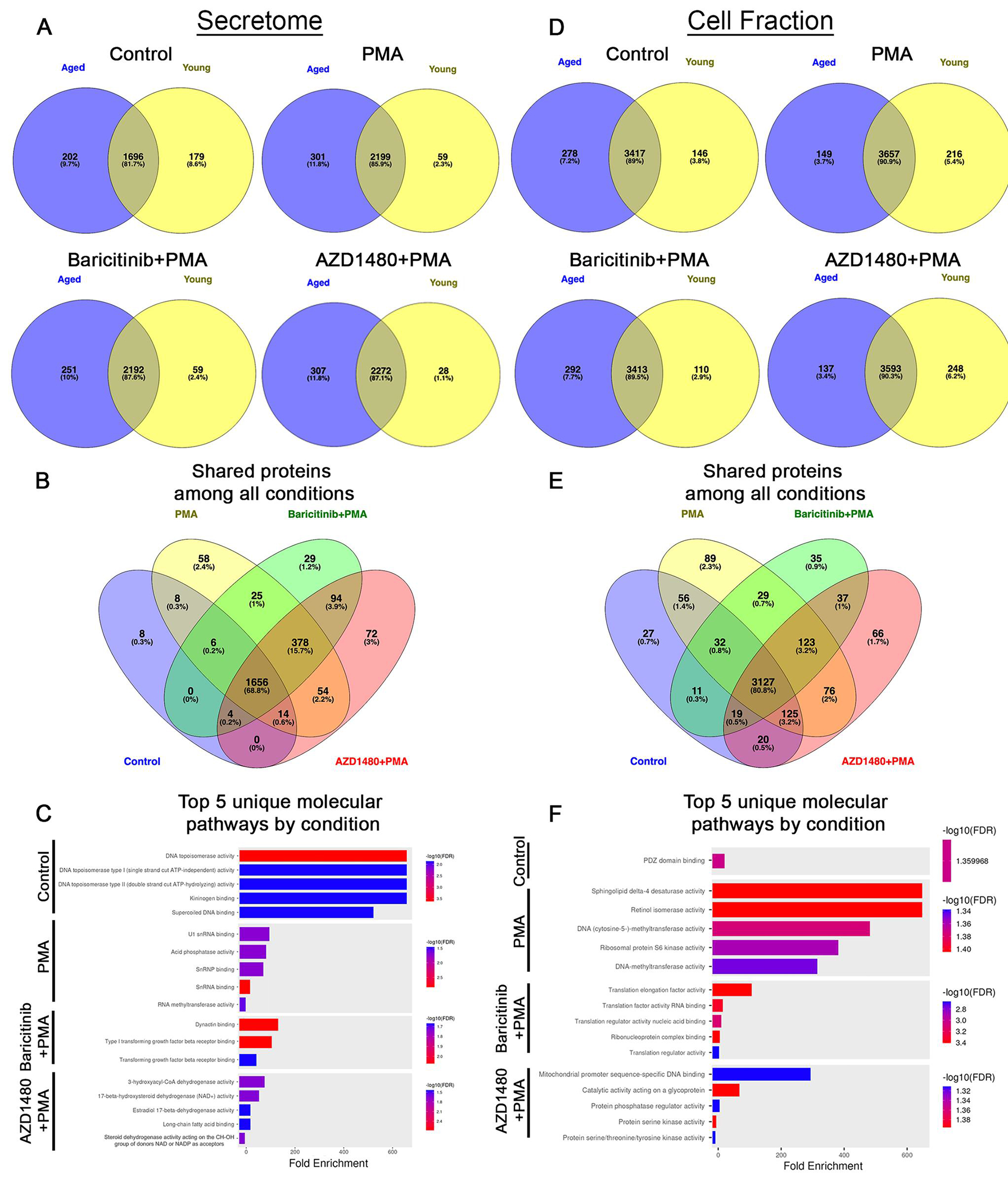
Age and JAK inhibition change protein profiles in neutrophils. (A) Venn diagrams representing unique and shared proteins identified in the secretome across different experimental conditions (control, PMA, baricitinib+PMA, and AZD1480+PMA) in neutrophils from young and aged mice. (B) Venn diagram representing shared secretome proteins across conditions. (C) Top 5 molecular pathways associated with uniquely expressed proteins in the secretome of each condition. (D) Venn diagrams illustrating unique and shared proteins identified in the cell fraction under different experimental conditions. (E) Venn diagram showing shared cell fraction proteins across conditions. (F) Top 5 molecular pathways associated with uniquely expressed proteins in the cell fraction of each condition.

After postprocessing, we compared the protein profiles of young and aged mice to identify proteins that were shared or uniquely associated with each condition, independent of age. Venn diagrams were generated using Venny (v2.1), a tool designed to compare shared and unique elements across datasets (https://bioinfogp.cnb.csic.es/tools/venny/). We used ShinyGO v0.82 to analyze uniquely expressed proteins for each condition (as shown in Fig. 1B, E) and identify associated Gene Ontology molecular pathways (https://bioinformatics.sdstate.edu/go/).

### Functional glycolysis (metabolic) assay

Neutrophils were isolated and incubated with the inhibitors as described above. Cells were resuspended in Seahorse XF RPMI media, supplemented with 2mM glutamine, and seeded at a density of 2×10^5^ cells/well at 50 μL in XF96-well plates (Agilent, catalog #103794-100) coated in Cell-Tak adhesive (Corning, catalog #354240). Plates were then centrifuged at 200×*g* for 1min and allowed to incubate in a non-CO_2_ incubator. Plates were then analyzed by the Seahorse Bioscience XF Pro instrument, which measures the extracellular acidification rate over time. Sequential injections of glucose, oligomycin, and 2-deoxy-d-glucose were injected at 10mM, 1.5μM, and 100mM, respectively. Assays were run for 1h 45 min.

### Luminex (ELISA)

The 22-plex Luminex assays (Bio-techne, CUSTOM-LXSA-M22) were run using supernatants collected for mass spectrometry. The proteins measured were proinflammatory cytokines (IL-1β, IL-1α, IL-6, IL-17, TNF-α, IFN-γ), anti-inflammatory cytokines (IL-10), chemokines (CCL2, CXCL1, CXCL2, CXCL10, SDF-1α), growth factors (G-CSF, insulin-like growth factor 1 [IGF-1], vascular endothelial growth factor [VEGF]), proteases (S100A8, Serpin E1, MMP-8), and complement receptor (C1qR). Luminex assays were run according to the manufacturer’s protocol by the Flow Cytometry and Single Cell Core facility at the WVU Health Science Center. Assays were run on the Luminex MAG PiX instrument using the xPONENT software v4.3 update 1.

### Statistical analyses

Data from blinded unbiased stereological counts and migration assays were statistically analyzed using one-way or 2-way analysis of variance (ANOVA), evaluating interactions between condition and age. For the metabolism assay, raw data were collected, and the area under the curve (AUC) was calculated for each group. AUC values were compared using one-way or 2-way ANOVA, followed by Tukey post hoc analysis. Mass spectrometry data were analyzed by generating functional pathway and protein-level heat maps, with positive and negative *z*-scores indicating up- and down-regulation, respectively. Fisher exact test was employed as a post hoc analysis for heatmaps. For individual protein analyses, intensity values from each animal were subjected to 2-way ANOVA to assess the effects of condition and age on protein abundance, with Tukey post hoc tests used to identify significant group differences. All data are presented as mean ± standard error of the mean (SEM), with statistical significance set at *P*<0.05. Statistical analyses were conducted using GraphPad Prism v10.4 software.

## Results

Neutrophils undergo an aging process after release from the bone marrow.^23^ The length of temporal aging is species dependent. For example, mouse neutrophils have a lifespan of approximately 18h and those from humans 5.4d.^24^ Beside the temporal age of neutrophils,^23^ the biological age of the host influences the grade of cells released from the progenitor cells in the bone marrow. In this study, we investigate the effect of biological aging on neutrophil function under normal conditions. For the remainder of this article, the term “young neutrophils” refers to neutrophils isolated from young mice (3mo) and “aged neutrophils” to neutrophils isolated from aged mice (≥22 mo). To control for the temporal age, all isolations of neutrophils were conducted at the same time each day. The isolation protocol used, MojoSort negative selection kit from BioLegend, enriched neutrophils in our samples from 40% to 80% (Fig. S1A). Further characterization showed no differences in cell morphology, cell diameter, or viability of isolated neutrophils between the aged or young neutrophils in any of the conditions (Fig. S1B, C). Viability was further confirmed by calculating cell density, using DAPI staining, in both age groups and all conditions (Fig. S1D).

It is well established that PMA modulates neutrophil function by activating various components of the protein kinase C (PKC) family of proteins. Through its effect on PKC-α, -βI, -βII, and -δ,^25^ PMA induces neutrophil migration^26^ and NADPH oxidase (NOX) formation.^27,28^ In this study, to determine which neutrophil functions require early JAK modulation, neutrophils from young and aged mice were exposed to a pan-JAK inhibitor (baricitinib^29^) or a JAK2 selective inhibitor (AZD1480^30^) followed by a low dose of PMA (20ng/mL or 32.43nM). This PMA concentration requires longer incubation periods to elicit neutrophil effector functions. Validation experiments showed that this concentration led to migration at 120min, degranulation at 150min, and NETosis at 180min as previously described in neutrophils from young mice^31,32^ (Fig. S1E, F, I). Of note, all experiments below were conducted at a shorter incubation period to characterize early cellular changes under all conditions.

There is little known of the regulation of early intracellular changes in neutrophils upon activation. To assess these changes, neutrophils were incubated with a low PMA dose for 45min prior to secretome (Luminex and mass spectrometry) and proteomic (mass spectrometry) assessment. Additional control experiments were conducted to characterize the specificity and off-target effect of the JAK inhibitors (Fig. S1). To assess the specificity of the drugs used, G-CSF, a proven JAK2 activator, was added to neutrophil cultures.^19,33^ G-CSF significantly increased neutrophil migration in the absence of PMA (Fig. S1G). The presence of baricitinib (*P*=0.017) and AZD1480 (*P*=0.046) abolished the effect of G-CSF on neutrophil migration, confirming the specificity of both inhibitors (Fig. S1G). To assess off-target effect, drugs were added to neutrophils from young mice without PMA. Our data suggest that neither baricitinib nor AZD1480 alone had an effect on neutrophil degranulation or migration (Fig. S1H, I).

Proteomic analysis of the neutrophil secretome revealed distinct protein populations in neutrophils from young male and female mice under all conditions, which was lost with age (Fig. S1K). Analysis of the cellular protein fraction showed little divergence across groups (Fig. S1K).

### JAK2 inhibition changes the proteomic profile of neutrophils with age

Mass spectrometry data were organized to determine uniquely expressed and shared proteins in all conditions. The results revealed that 2,765 unique proteins were present in the secretome and 4,273 in the cell fraction. Treatment/condition-specific proteins were also found in each compartment (Fig. 1A, D). Several proteins were expressed in all groups and conditions (Fig. 1B, E). Unique proteins were detected in all conditions (Fig. 1B, E).

#### Secretome

Under control conditions, we identified 179 uniquely expressed proteins in young neutrophils and 202 in aged neutrophils, with 1,696 (81.7%) proteins shared between the 2 groups (Fig. 1A). The addition of PMA changed the number of uniquely expressed proteins to 59 and 301 in the young and aged neutrophils, respectively, and increased the number of shared proteins across groups to 2,199 (85.9%). Compared to PMA, inhibition of all JAK proteins with PMA activation (baricitinib+PMA) decreased the number of uniquely expressed proteins in aged neutrophils to 251, while the young neutrophils had same number of unique proteins (59), and slightly decreased the number of shared proteins to 2,192 (87.6%). Compared to PMA, inhibition of JAK2 alone with PMA activation (AZD1480+PMA) increased the number of unique proteins in the aged neutrophils (307), decreased the number of unique proteins (28) in the young neutrophils, and increased shared proteins to 2,272 proteins (87.1%) (Fig. 1A). Direct comparisons of the shared proteins across all conditions showed 1,656 (68.8%) common proteins, with 8 proteins unique to control, 58 proteins unique to PMA, 29 to baricitinib+PMA, and 72 to AZD1480+PMA (Fig. 1B). Of the uniquely expressed protein in each condition, we highlight the top 5 unique molecular pathways. The secretome of neutrophils under control conditions contained proteins involved in DNA remodeling. With PMA stimulation, neutrophil secretome is enriched with spliceosome components and RNA-modifying enzymes, which can regulate host gene expression and disrupt viral replication.^34^ With pan-JAK inhibition (baricitinib+PMA), the secretome contained proteins that modulates the TGF-β pathway. With JAK2 inhibition (AZD1480+PMA), secretome contained enzymes involved in lipid and steroid metabolism (Fig. 1C).

#### Cell fraction

Under control conditions, 146 uniquely expressed proteins were present in young neutrophils, 278 in aged neutrophils, with 3,417 (89%) proteins shared between the 2 groups (Fig. 1D). The addition of PMA changed the number of uniquely expressed proteins to 216 and 149 in the young and aged neutrophils, respectively, and increased the number of shared proteins to 3,657 (90.9%). Compared to PMA, inhibition of all JAK proteins (baricitinib+PMA) decreased the number of uniquely expressed proteins to 110 in young neutrophils, increased to 292 in age neutrophils, and decreased the number of shared proteins to 3,413 (89.5%). Compared to PMA, inhibition of JAK2 alone (AZD1480+PMA) increased the number of unique proteins in the young neutrophils to 248, decreased the number of unique proteins in aged neutrophils to 137, and decreased the number of shared proteins to 3,593 (90.3%) (Fig. 1D). Direct comparisons of the shared proteins across all conditions showed 3,127 (80.8%) common proteins, with 27 proteins unique to control, 89 to PMA, 35 to baricitinib+PMA, and 66 to AZD1480+PMA (Fig. 1E). Similar to the secretome, we highlight the top 5 unique molecular pathways present in the cell fraction of each condition (Fig. 1F). Under control conditions, unique proteins expressed in neutrophil cell fraction were those important for PSD-95/Discs Large/ZO-1 domain binding. With PMA stimulation, unique pathways relate to the activity of enzymes involved in lipid remodeling, epigenetic modification, and protein synthesis. With pan-JAK inhibition (baricitinib+PMA), enrichment was in elongation factors and RNA-binding proteins. With JAK2 inhibition (AZD1480+PMA), pathways associated with the regulation of mitochondrial gene expression and intracellular signaling through phosphorylation and glycoprotein modification were enriched (Fig. 1F). These results show that JAK proteins can have unique effects on molecular pathways in neutrophil. Overall, these results highlight the shared and distinct proteins associated with neutrophil activation that are both dependent and independent of the JAK signaling pathway. To understand the effect of JAK inhibition on neutrophil functions with age, the remainder of this article will focus solely on shared proteins in the cell fraction (~73% of all detected proteins) and secretome (~68% of all detected proteins).

### JAK2 regulates proteins involved with neutrophil migration in an age-dependent manner

To determine how JAK activation contributes to neutrophil migration with age, JAK signaling was inhibited in neutrophils isolated from young (young neutrophils) and aged (aged neutrophils) mice, activated by PMA, placed in Boyden chambers (with 3-μm pores), and assessed for migration after 120min of incubation. Cell migration is the result of physiological changes associated with intracellular modification, including cytoskeletal remodeling to form pseudopodia (cell fraction and migration analysis), in response to various extracellular cues (secretome and cytokine analysis). To characterize early cellular changes that precede migration, a subset of isolated neutrophils was incubated with inhibitors and activated with PMA for 45min. The supernatant was used to analyze the secretome to determine the effect on cellular response to the inhibition; the cell pellet was analyzed as the cell fraction to determine the effect of the inhibition on intracellular signaling.

#### Secretome

**Figure 2.**
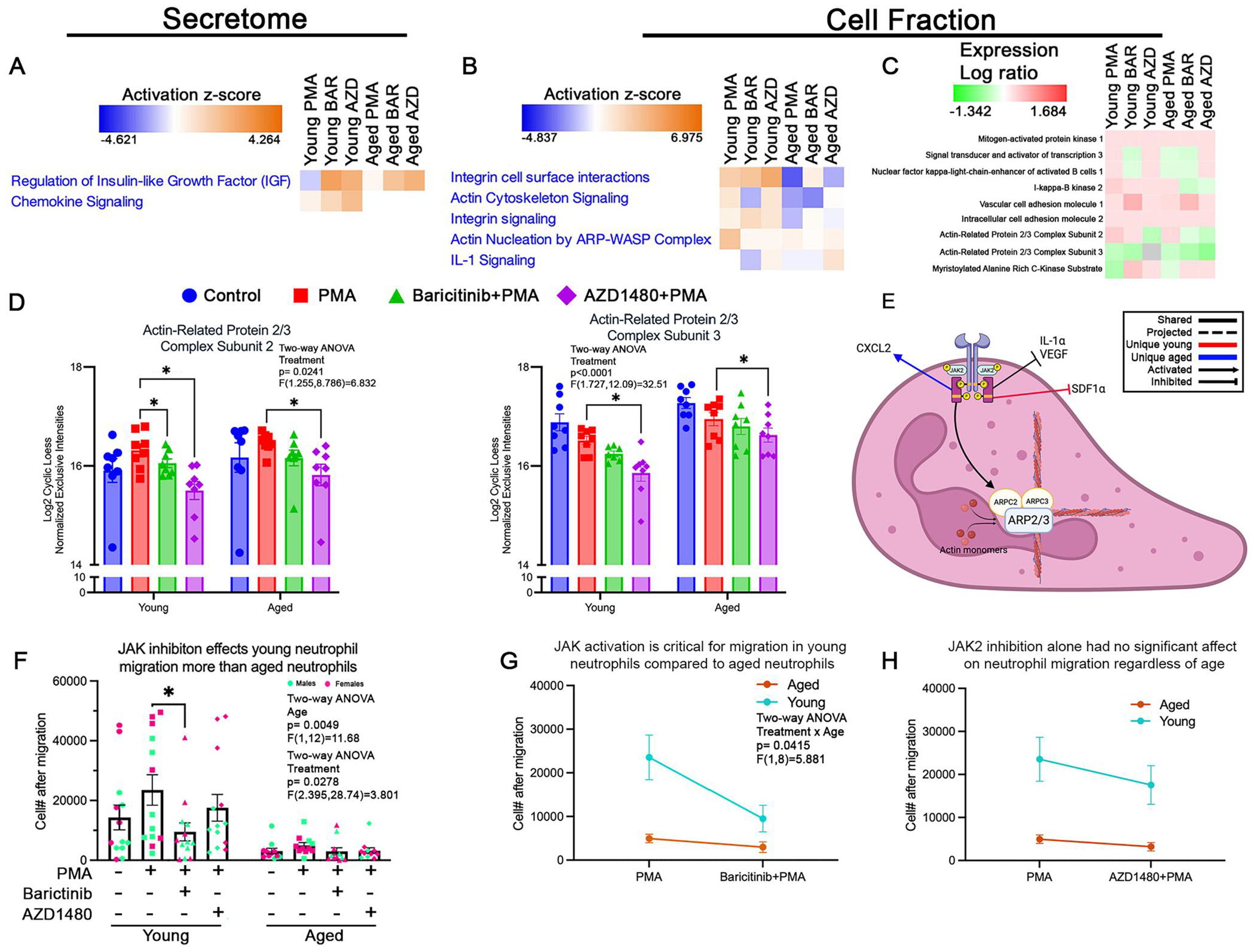
Age-specific changes in JAK2 regulation of neutrophil migration. (A) Functional heatmap of select pathways in secretome of neutrophils from young and aged mice across conditions (normalized to control). Heatmap show *z*-scores generated from *P* values and log fold/ratio changes. JAK inhibition (baricitinib [BAR], pan-JAK; AZD1480 [AZD], JAK2) increased regulation of IGF and chemokine signaling pathways compared to PMA treatment in neutrophils from young mice. In neutrophils from aged mice, both AZD and BAR treatment only increased regulation of IGF compared to PMA treatment. (B) Functional heatmap of migration-associated pathways in the cell fraction of samples. AZD treatment (JAK2 inhibition) led to an increase in integrin signaling, IL-1 signaling, and actin cytoskeleton signaling, and a decrease in actin nucleation by ARP-WASP compared to PMA treatment in neutrophils isolated from young mice. In neutrophils from aged mice, JAK2 inhibition increased all listed functions compared to PMA treatment, except for actin nucleation by ARP-WASP. BAR treatment (pan-JAK inhibition) led to an increase in integrin signaling and a decrease in the rest of the listed functions compared to PMA treatment in neutrophils from young mice. BAR-treated neutrophils from aged mice increased integrin signaling and decreased actin cytoskeleton signaling and actin nucleation by ARP-WASP, with no changes in IL-1 signaling compared to PMA-treated cells. (C) Heatmap depicting abundance of protein (log ratio normalized to control), associated with neutrophil motility. Overall, JAK regulation on neutrophil motility proteins is age dependent. AZD-treated neutrophils from young and aged mice showed a decrease in ARPC2 and an increase in myristoylated alanine-rich c-kinase substrate. AZD-treated neutrophils from aged mice had a decrease in I-κB kinase. BAR-treated neutrophils from young and aged mice showed increased VCAM1. BAR-treated neutrophils from young mice display decreased STAT3 and NF-κB. Those from aged mice showed a decrease in ARPC2 and I-κB kinase. (D) Bar graphs showing the relative abundance of 2 cytoskeletal proteins in each condition. ARPC2 and ARPC3 levels were decreased in neutrophils from young and aged mice in the presence of AZD (JAK2 inhibition). In neutrophils from young mice, BAR (pan-JAK inhibition) also decreased ARPC2 levels. (E) Proposed model of molecular mechanism of JAK2 modulation of neutrophil migration. We propose that JAK2 promotes the assembly of the actin nucleation complex by increasing the levels of ARPC2/3. This leads to actin branching and formation of pseudopodia to facilitate movement. Age-dependent regulation of migration by JAK2 occurs through its differential modulation of neutrophil chemokine release (increased CXCL2 in neutrophils from aged mice, and decreased SDF-1α in neutrophils from young mice). (F) Functional assessment of migration showed that overall, fewer neutrophils from age mice migrate toward IL-8 compared to those from young mice. Only BAR significantly decreased neutrophil migration of neutrophils from young mice compared to PMA treatment. (G, H) Migration pattern between neutrophils from young and aged mice after pan-JAK or JAK2 inhibition (young: *n*=13/condition; aged: *n*=11/condition). *P<0.05; bar graphs represent mean±SEM. (D, F) Individual points correspond to individual mice (pink, females; green, males). Diagram generated with BioRender.

The secretome data showed that JAK signaling is important for the release of extracellular factors that mediate migration in neutrophils. The IGF pathway activity was regulated by JAK2, as seen by the increased proteins in this pathway under JAK2 inhibition (AZD1480) in young and aged neutrophils (Fig. 2A). IGF plays a crucial role in plasma membrane dynamics by promoting lipid incorporation into the plasma membrane, which increases membrane protrusion to form filopodia and increase motility.^35^ Chemokine signaling, essential for directing neutrophil migration, also showed JAK2-dependent modulation. In young neutrophils, JAK2 inhibition had no effect on CXCL2 release, while decreasing its release in aged neutrophils (*P*=0.0279; Fig. S2). IL-1α, a proinflammatory cytokine that enhances neutrophil recruitment and activation, is upregulated in both young (*P*=0.0004) and aged (*P*=0.036) neutrophils following JAK2 inhibition (Fig. S2). The secretion of VEGF, a potent angiogenic factor, increased only in aged neutrophils following JAK2 inhibition (*P*<0.0001; Fig. S2). SDF-1α secretion increased only in young neutrophils with JAK2 inhibition (*P*=0.022; Fig. S2). These findings suggest that JAK2 inhibition differentially regulates neutrophil secretion of key proteins in an age-dependent manner.

Interestingly, our data showed that the proteins modulated by JAK2 inhibition alone were also inhibited by pan-JAK inhibition, including IGF, IL-1α, SDF-1α (young only), and VEGF. These data suggest that JAK2 may have the most direct effect on the regulation of these proteins. There were a few pathways that were unique to the pan-JAK modulation. SDF-1α secretion was significantly increased in aged neutrophils following pan-JAK inhibition (*P*=0.0196; Fig. S2). C1qR a complement receptor promoting neutrophil migration, was significantly elevated in young (*P*=0.0049) and aged (*P*<0.0001) neutrophils following pan-JAK inhibition (Fig. S2).

#### Cell fraction

Actin cytoskeleton rearrangement is crucial for neutrophil migration. In young and aged neutrophils, actin cytoskeleton signaling was influenced by JAK2 activation, as seen by the downregulation of the Actin-related protein (ARP)-Wiskott-Aldrich syndrome protein (ARP-WASP) complex, a key driver of actin remodeling (Fig. 2B).^36^ Actin-related protein complex subunits 2 and 3 (ARP2/3), which is essential for cytoskeletal rearrangement,^37^ was decreased by JAK2 inhibition in young (ARPC2, *P*=0.0125; ARPC3, *P*=0.022; Fig. 2D) and aged (ARPC2, *P*=0.018; ARPC3, *P*=0.0474; Fig. 2D) neutrophils. The myristoylated alanine-rich protein kinase C (MARCKS) pathway, a critical regulator of ARP2/3 phosphorylation and actin nucleation,^38,39^ was upregulated by JAK2 inhibition in both young and aged neutrophils (Fig. 2C). In aged neutrophils, JAK2 inhibition increased NF-κB1, which regulates neutrophil migration through MAPK signaling^40^ (Fig. 2C). I-κB kinase 2, responsible for phosphorylating MAPK, decreased with JAK2 inhibition in aged but not young neutrophils (Fig. 2C). These findings suggest that JAK2 signaling predominantly regulates migration through cytoskeletal remodeling in both young and aged neutrophils (Fig. 2E).

Pan-JAK inhibition showed an interesting regulatory pattern in terms of modulating neutrophil migration. In aged neutrophils, integrin cell surface interactions were upregulated by pan-JAK inhibition (Fig. 2B). IL-1 signaling, known to influence neutrophil migration via CXCR2,^41^ was decreased by pan-JAK inhibition in young neutrophils, with no effect in aged neutrophils (Fig. 2B). NF-κB1 was decreased with pan-JAK inhibition only in young neutrophils (Fig. 2C). I-κB kinase 2 was decreased with pan-JAK inhibition only in aged neutrophils (Fig. 2C). These data suggest that other JAK proteins can directly modulate neutrophil migration towards IL-8.

Our functional assessment of neutrophil migration, using the Boyden chamber and IL-8, corroborated the data in the secretome and cell fraction mass spectrometry analyses with pan-JAK inhibition, leading to fewer migrating cells in young neutrophils (*P*=0.036; Fig. 2F). JAK inhibition had no effect on the migration of aged neutrophils (Fig. 2F). A direct comparison of the young and aged neutrophils showed more migration by young neutrophils compared to aged neutrophils (pan-JAK: *P*=0.0191; JAK2: *P*=0.0169; Fig. 2F, G).

### JAK2 regulates degranulation in young neutrophils only

Degranulation in neutrophils is the release of granule content (antimicrobial proteins, enzymes, ROS, etc.) into the extracellular space. This tightly regulated process plays a pivotal role in innate immunity.^42^ Neutrophil degranulation is a process often associated with the release of tertiary (gelatinase), secondary (specific), and primary (azurophilic) granules. With primary granules containing potent enzymes like myeloperoxidase (MPO), NOX, and neutrophil elastase (NE), the threshold of release is higher due to their toxic effects.^43^ Secondary granules store lactotransferrin (LTF) and lipocalin-2 (LCN-2) to limit bacterial growth,^44^ and tertiary granules contain MMPs to break down extracellular matrices.^45^ The fusion of neutrophil granules with the plasma membrane is mediated by SNARE proteins, including syntaxins, vesicle-associated membrane proteins, and synaptosomal-associated proteins, which coordinate vesicle docking and membrane fusion for efficient secretion of antimicrobial contents.^46^ Calcium signaling plays a crucial role in neutrophil degranulation by activating actin cytoskeletal remodeling and triggering granule transport by motor proteins.^47^

#### Cell fraction

**Figure 3.**
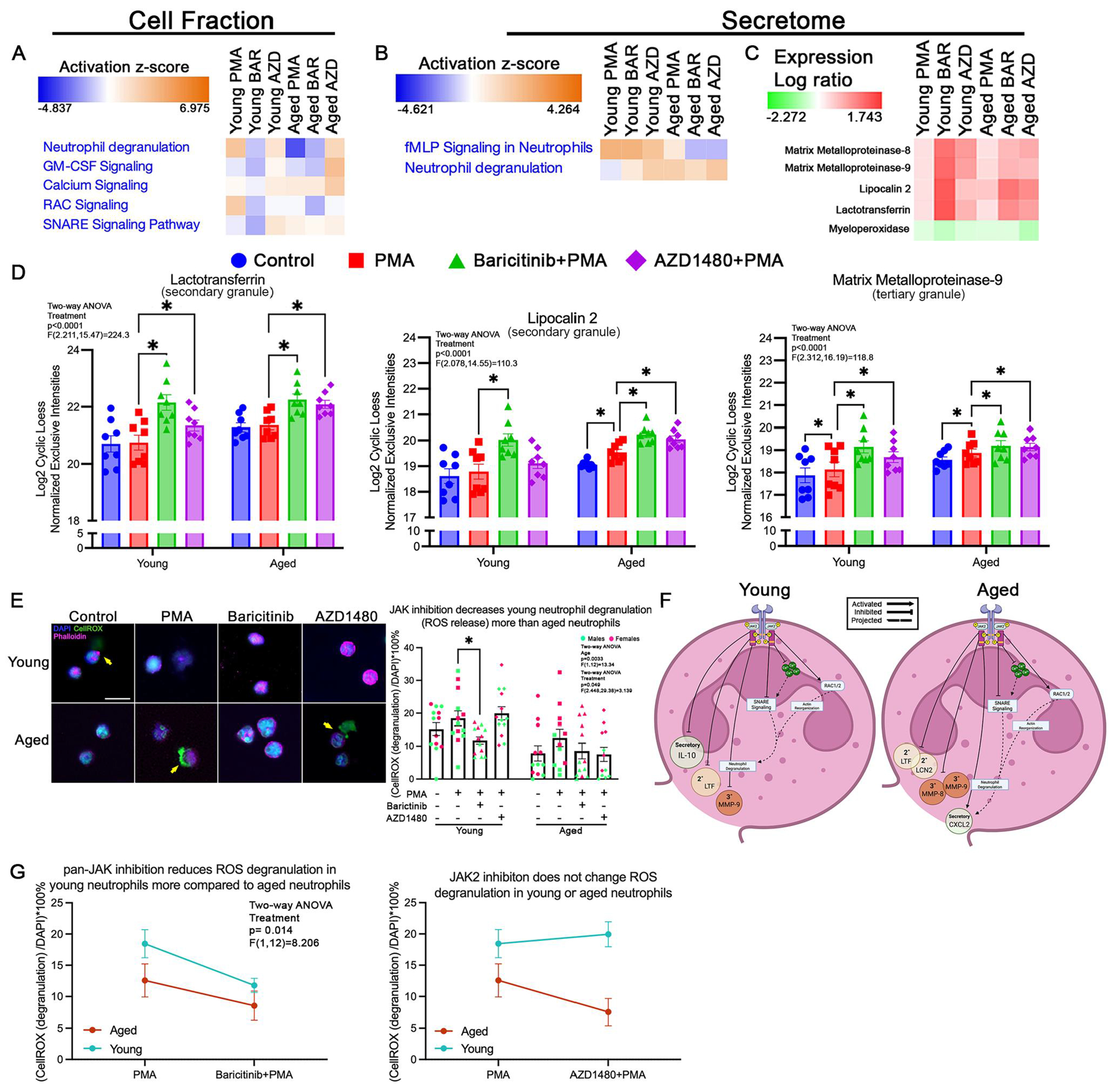
JAK2 signaling differentially regulates degranulation in neutrophils from young and aged mice. (A) Functional heatmap of select pathways in cell fraction revealed that AZD1480 (AZD)–treated neutrophils from young mice had increased activity in GM-CSF, SNARE, and calcium signaling, and decreased activity in neutrophil degranulation and RAC signaling pathways compared to PMA. AZD-treated neutrophils from aged mice increased neutrophil degranulation, GM-CSF, and calcium signaling pathways, with no changes in RAC and SNARE signaling compared to PMA. Baricitinib (BAR)–treated neutrophils from young mice had decreased signaling in all listed pathways compared to PMA. BAR-treated neutrophils from aged mice had decreased GM-CSF, SNARE, and RAC signaling, increased neutrophil degranulation, and no change in calcium signaling compared to PMA. (B) Functional heatmap of the secretome showed an increase in neutrophil degranulation pathway only in neutrophils from young mice with AZD and BAR treatment compared to PMA treatment. In neutrophils from aged mice, AZD and BAR decreased fMLP signaling pathways compared to PMA treatment, with no effect on the neutrophil degranulation pathway. (C) Heatmap depicting protein abundance showed that AZD and BAR increased the release of granule proteins. (D) Bar graphs displaying relative abundance of secondary and tertiary granule proteins, LTF, LCN-2, and MMP-9 across experimental conditions. In neutrophils from young mice, both AZD and BAR increased LTF and MMP-9. However, LCN-2 was only increased with BAR in neutrophils from young mice. In neutrophils from aged mice, BAR and AZD increased the release of LTF, LCN-2, and MMP-9. (E) Representative images of primary granule degranulation (CellROX green reagent, adjacent to phalloidin [pink] delineated cells; yellow arrows). Quantification of images showed that under control, PMA, and AZD conditions, neutrophils from young mice degranulate (release ROS) more than neutrophils from aged mice. BAR treatment decreased the number of degranulating (release ROS) neutrophils from young mice. (F) Proposed model of JAK2 regulation of neutrophil degranulation with age. We propose that in neutrophils from young and aged mice, JAK2 activation inhibits the release of secondary and tertiary granules. In neutrophils from aged mice, JAK2 activation also promotes the release of secretory vesicle content. We postulate that this modulation is mediated by JAK2 regulation of calcium signaling and SNARE complex activation. (G) Differences in degranulation pattern between neutrophils from young and aged mice showed that BAR (pan-JAK inhibition) decreased the proportion of neutrophils from young mice that degranulate. AZD1480 (JAK2 inhibition) treatment had no effect on the proportion of neutrophils from young mice and aged mice that degranulate (young: *n*=13/condition; aged: *n*=13/condition). Scale bar=20 μm; *P<0.05; bar graphs represent mean±SEM. (D, E) Individual points correspond to individual mice (pink, females; green, males). Diagram generated with BioRender.

Granulocyte-macrophage colony-stimulating factor (GM-CSF) signaling, known to enhance degranulation through activation of PI3K/AKT and MAPK,^48,49^ was upregulated in both young and aged neutrophils following JAK2 inhibition (Fig. 3A). Ras-related C3 botulism toxin substrate (RAC) signaling decreased in young and aged neutrophils with JAK2 inhibition (Fig. 3A). Inhibition of RAC activity disrupts granule fusion and attenuates the release of antimicrobial mediators.^50^ Intracellular calcium signaling was elevated in young and aged neutrophils following JAK2 inhibition (Fig. 3A). Similarly, soluble N-ethylmaleimide-sensitive-factor attachment protein receptor (SNARE) signaling, which regulates the fusion of granules to the plasma membrane, was upregulated in both age groups (Fig. 3A). Pan-JAK inhibition decreased the activity of several proteins involved in neutrophil degranulation. In young neutrophils, pan-JAK inhibition reduces GM-CSF, calcium, RAC, and SNARE signaling (Fig. 3A). In aged neutrophils, pan-JAK inhibition decreased GM-CSF and RAC signaling pathways (Fig. 3A). The fact that JAK2 inhibition increased protein levels while pan-JAK inhibition decreased protein level suggests that each JAK protein differentially modulates neutrophil degranulation.

#### Secretome

In aged neutrophils, JAK2 inhibition decreased the activity of the N-formyl-methionyl-leucyl-phenylalanine (fMLP) signaling pathway (Fig. 3B). Our data show that PMA stimulation significantly upregulates both LCN-2 (*P*=0.025) and MMP-9 (*P*=0.013) in aged neutrophils compared to control, whereas in young neutrophils with PMA stimulation, only MMP-9 was increased relative to control (*P*=0.031; Fig. 3C). Characterization of tertiary granule protein release revealed that MMP-9 levels were significantly increased with JAK2 inhibition, in young (*P*=0.018) and aged (*P*=0.003) neutrophils (Fig. 3C). Additionally, MMP-8 levels increased following JAK2 inhibition (*P*=0.04) in aged neutrophils (Fig. S2). Secondary granule proteins LTF and LCN2 were both increased with JAK2 inhibition (Fig. 3C). In aged neutrophils, both LTF (*P*<0.0001) and LCN2 (*P*=0.0001) were increased (Fig. 3D). In young neutrophils, only LTF was increased with JAK2 inhibition (*P*=0.0023; Fig. 3D). MPO (a primary granule protein) was not affected by JAK2 inhibition (Fig. 3C). Cytokine release, associated with neutrophil secretory vesicles, was regulated in an age-dependent manner. JAK2 inhibition increased the release of anti-inflammatory cytokine IL-10 (*P*=0.0413) in young neutrophils (Fig. S2) and decreased CXCL2 in aged neutrophils (Fig. S2).

Pan-JAK inhibition had the same effect on fMLP and MMP-9 in young and aged neutrophils as JAK2 inhibition, suggesting that JAK2 may be driving the changes observed in these pathways (Fig. 3B, C). MMP8 levels increased in young neutrophils after pan-JAK inhibition (*P*=0.009; Fig. S2). LTF and LCN2 increased with pan-JAK inhibition (Fig. 3C, D). MPO was not affected in young or aged neutrophils with pan-JAK inhibition (Fig. 3C). Analysis of cytokine secretion revealed that pan-JAK inhibition significantly increased secretion of TNF-α (*P*=0.0025) and CXCL10 (*P*=0.0114) in aged neutrophils (Fig. S2). IL-10 (*P*=0.0089) and CXCL10 (*P*=0.0001) levels were elevated in young neutrophils under pan-JAK inhibition (Fig. S2). These findings suggest that early events drive differential granule release in an age-dependent manner and that multiple JAK proteins may be responsible for this. Moreover, regardless of age, JAK2 activation inhibits the release of secondary and tertiary granules with no effect on the release of primary granule content (Fig. 3E). Our data also indicate that the release of secretory vesicle may be differentially regulated by various JAK proteins.

Functional assessment of degranulation was done by characterizing the release of ROS, a functional assessment of primary granule function, in the extracellular space. Cells with adjacent CellROX signal, immediately outside the phalloidin stain (Fig. 3E; yellow arrow), were identified as degranulating. Only pan-JAK inhibition significantly reduced ROS release in young neutrophils (*P*=0.034), with no effect on ROS release in aged neutrophils (Fig. 3E). Altogether, these data suggest that although primary granule release is not mediated by JAK activation, ROS release is dependent on JAK activity in neutrophils from young mice, with age-dependent release of secretory vesicles (Fig. 3F).

### JAK2 inhibition alone did not affect NET formation in neutrophils

To date, the only studies examining JAK2 signaling in relation to NETosis have focused on the gain-of-function JAK2 V617F mutation in myeloproliferative neoplasia.^51^ This study showed that the JAK2 mutation increases NETosis. In this work, we sought to determine whether nonmutated JAK proteins also contribute to NETosis under normal conditions. NET production was assessed using 2 different methods, IHC and free DNA. IHC assessment of NETs (DAPI^+^Histone1^+^MPO^+^) showed no effect of JAK inhibition (Fig. S3A). However, measuring free DNA, using PicoGreen, in the supernatant showed that PMA alone increased the amount of free DNA in the samples, as expected (Fig. S3B) in aged neutrophils. Only pan-JAK inhibition decreased the levels of free DNA from the PMA control (Fig. S3B) in aged neutrophils.

Proteomic analysis of the cell fraction showed that the NET signaling and IL-17 signaling pathways were increased by JAK2 inhibition in young neutrophils. The NET signaling, IL-1 signaling, and IL-17 signaling pathways were increased with JAK2 inhibition in aged neutrophils (Fig. S3C). NE was increased by JAK2 inhibition compared to PMA in young neutrophils (*P*=0.013), but in aged neutrophils NE was unchanged across conditions (Fig. S3E). MPO was unchanged across conditions in both young and aged neutrophils with JAK2 inhibition. NOX1, a critical component of ROS-dependent NETosis, was significantly decreased following JAK2 inhibition compared to PMA in both young (*P*=0.002) and aged (*P*=0.025) neutrophils (Fig. S3E). There was no change in peptidylarginine deiminase 4 (PAD4) levels in young or aged neutrophils under any conditions (Fig. S3E).

Pan-JAK inhibition led to an increase in NETosis signaling pathway and decreases in IL-27 and IL-1 signaling compared to PMA in young neutrophils (Fig. S3C). Whereas in aged neutrophils, pan-JAK inhibition led to an increase in IL-27 signaling, and no effect in NETosis and IL-1 signaling compared to PMA (Fig. S3C). MPO abundance is significantly decreased compared to PMA (*P*=0.024) in aged neutrophils (Fig. S3E). Similar to JAK2 inhibition, NOX1 is significantly decreased compared to PMA in both young (*P*=0.003) and aged (*P*=0.0002) neutrophils (Fig. S3E). Taken together, the early protein changes suggest that JAK proteins work in concert to modulate aspects of NET release by regulating NOX1, MPO, and NE levels, key proteins involved in NET formation.

### JAK2 regulates the shift in glucose metabolism with age

**Figure 4.**
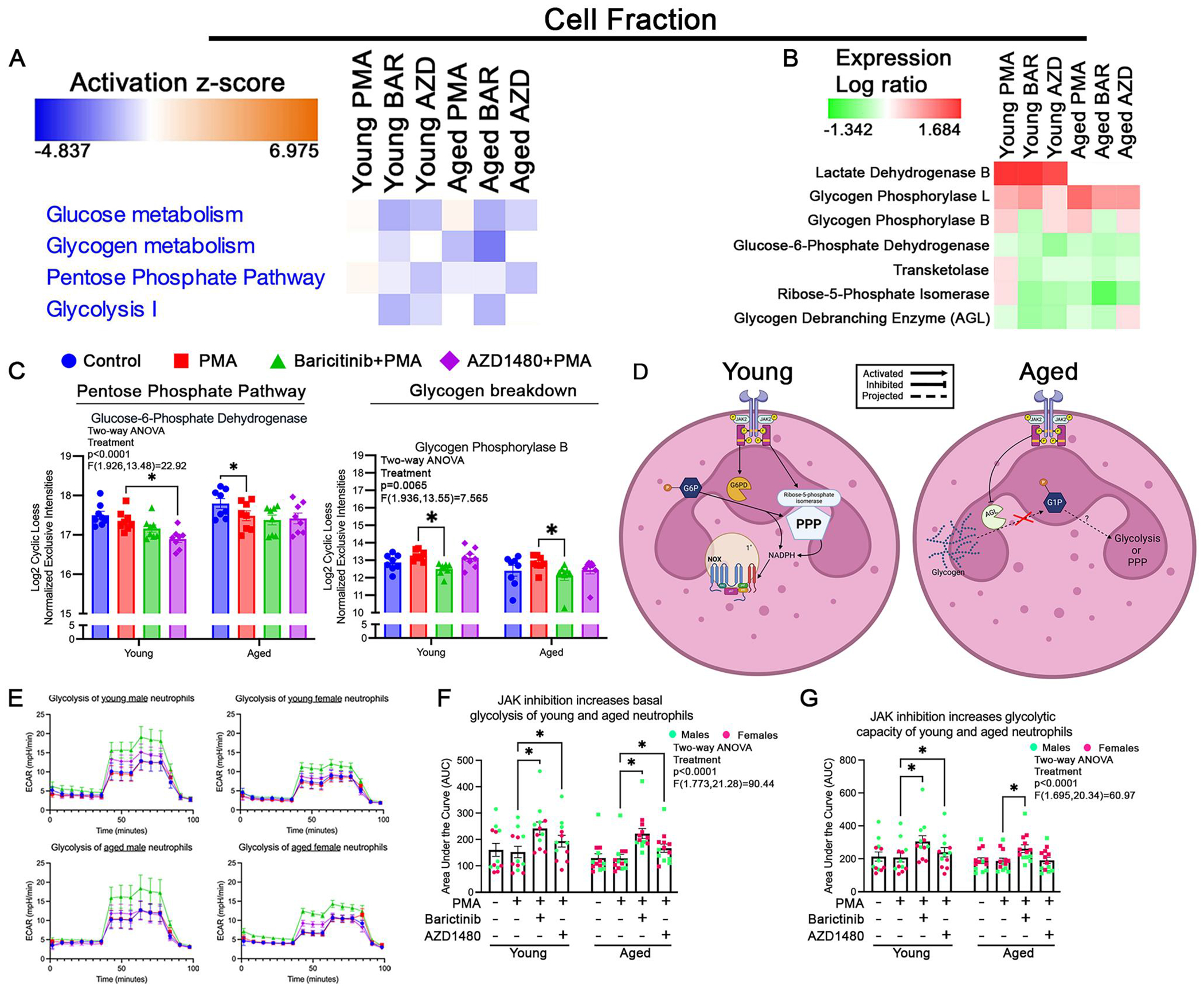
JAK2 modulation of neutrophil metabolism is age dependent. (A) Functional heatmap of cell fraction showed that AZD1480 (AZD) treatment decreased glucose metabolism, pentose phosphate pathway (PPP), and glycolysis I compared to PMA in neutrophils from young mice. AZD-treated neutrophils from aged mice decreased glucose metabolism and PPP, increased glycogen metabolism, and had no effect on glycolysis compared to PMA. Baricitinib (BAR) treatment decreased all pathways in neutrophils from both young and aged mice compared to PMA, except for the PPP in neutrophils from aged mice. (B) Heatmap of protein abundance showed that AZD and BAR treatment decreased protein abundance of all listed proteins in neutrophils from young mice compared to PMA, except for lactate dehydrogenase B and glycogen phosphorylase L. In neutrophils from aged mice, AZD treatment showed decreased abundance for glycogen phosphorylase L, an increase for glycogen debranching enzyme, and no change in the rest of the listed proteins. BAR treatment decreased all listed proteins in neutrophils from aged mice, except for lactate dehydrogenase B compared to PMA. (C) Bar graph showed that AZD significantly decreased levels of G6PD in neutrophils from young mice. BAR decreased the expression of glycogen phosphorylase B levels in neutrophils from young and aged mice. (D) Proposed model of JAK2 modulation of metabolism in neutrophils. We postulate that in neutrophils from young mice, JAK2 activity increases the activity of G6PD, which shuffles glucose into the PPP. This increase in activity leads to increased activity of ribose-5-phosphate isomerase and production of NADPH. In neutrophils from aged mice, JAK2 activation blocks the activation of AGL, which decreases the conversion of glycogen into glucose (G1P). (E) Representative histograms of Seahorse assay assessing glycolysis in neutrophils. ECAR is the acidification rate, ie lactate production, of cells under the various conditions (young: *n*=12/condition; aged: *n*=13/condition). (F, G) Quantification of ECAR using AUC of basal glycolysis (F) and glycolytic capacity (G) in neutrophils from young and aged mice. JAK inhibition increased basal glycolysis in both age groups. In neutrophils from young mice, JAK inhibition also increased glycolytic capacity. In neutrophils from aged mice, only BAR increased glycolytic capacity. *P<0.05; bar graphs represent mean±SEM. (C, F, G) Individual points correspond to individual mice (pink, females; green, males). Diagram generated with BioRender.

All neutrophil functions require energy, so the next cellular process we investigated was metabolism. Neutrophils primarily use glycolysis for energy generation.^52^ Neutrophil activation using PMA increases glucose shuffling into the PPP, which results in increased NADPH generation and oxygen consumption.^53^ Our data showed that PMA has no effect on glycolysis of young or aged neutrophils (Fig. 4) but, interestingly, increased PPP in young neutrophils and decreased it in aged neutrophils (Fig. 4A). These suggest that PMA effect on neutrophil metabolism is age dependent. PPP is a metabolic pathway that generates NADPH for biosynthetic reactions and ribose-5-phosphate for nucleotide synthesis. In neutrophils, PPP generation of NADPH is crucial to the generation of ROS by the NOX complex.^53^ Whether neutrophil metabolism is influenced by JAK signaling is yet unknown. In this study, we assessed the effect of JAK inhibitors on neutrophil metabolism by assessing glycolysis with the Seahorse XF analyzer (XF96) and characterizing the expression of metabolic proteins using mass spectrometry.

Glucose metabolism and PPP were decreased in young and aged neutrophils with JAK2 inhibition (Fig. 4A). Glucose-6-phosphate dehydrogenase (G6PD), a key enzyme in PPP, was significantly reduced (*P*=0.0415) in young neutrophils following JAK2 inhibition (Fig. 4B, C). Transketolase (TKT), an enzyme in the nonoxidative branch of the PPP, is responsible for converting intermediates from the oxidative phase into glycolytic substrates and generating NADPH. From our data, PMA increased TKT levels in young neutrophils only (Fig. 3B). JAK2 inhibition decreases TKT in young neutrophils, with no affect in aged neutrophils (Fig. 4B). Additionally, ribose-5-phosphate isomerase, a PPP enzyme involved in the conversion of G6P to ribulose 5-phosphate,^54^ decreased in young neutrophils following JAK2 inhibition (Fig. 4B). The activity of glycogen debranching enzyme (AGL), which facilitates glycogen breakdown, was increased in aged neutrophils under JAK2 inhibition (Fig. 4B). These findings suggest that JAK2 activity regulates PPP in young neutrophils and glycogen breakdown in aged neutrophils (Fig. 4D), suggesting an age-dependent switch in metabolic modulation.

Young neutrophils had decreased glucose metabolism, glycogen metabolism, PPP, and glycolysis following pan-JAK inhibition (Fig. 4A). Aged neutrophils had decreased glucose metabolism and glycolysis with pan-JAK inhibition (Fig. 4A). Lactate dehydrogenase B, critical for the reversible conversion of lactate to pyruvate, was increased in young neutrophils while aged neutrophils showed no change from control (Fig. 4B). The inactive “B” form of glycogen phosphorylase (PYGL), which converts glycogen into glucose-1-phosphate, was downregulated in both young (*P*=0.0049) and aged (*P*=0.0217) neutrophils following pan-JAK inhibition (Fig. 4B, C). These data suggest that combined JAK activation plays an important role in glucose metabolism in neutrophils.

Seahorse functional assessment of neutrophil glycolysis showed that JAK2 and pan-JAK inhibition increased basal and glycolytic capacity in young neutrophils (Fig. 4F, G). In aged neutrophils, JAK2 inhibition increased basal glycolysis, whereas pan-JAK inhibition increased both basal and glycolytic capacity (Fig. 4F, G). These data suggest that JAK inactivity leads to increased glycolysis and production of lactate. This is evident in young neutrophils, where functional assays and mass spectrometry data align, demonstrating a reduction in PPP-related signaling and enhanced glycolysis with JAK inhibition. We postulate that this would decrease glucose shuffling into PPP, thereby decreasing NADPH production, and the downregulation of NOX1 in neutrophils. Our data showed that JAK2 and pan-JAK inhibition decreased NOX1 in young neutrophils (Fig. S3E). In aged neutrophils, our data suggest that impaired glycogen breakdown correlates with increased lactate production. The increased lactate production could be due to an increased intake of extracellular glucose and increased glycolysis by these cells. Overall, these data suggest age-dependent shifts in JAK regulation of neutrophil metabolism.

## Discussion

Neutrophil effector functions and activity are widely studied. However, the regulation of the molecular mechanisms preceding these functions is largely unknown. The results of this study elucidate intracellular pathways and molecules in neutrophils that are dependent on JAK2 activation for migration, degranulation, and glycolysis.

JAK2 modulates neutrophil migration in response to IL-8 stimulation.^19^ Our findings revealed that the molecular mechanism that underlies this modulation is age dependent. In neutrophils, JAK2 mediated migration by modulating cytoskeletal remodeling by increasing levels of ARPC2 and ARPC3 in both young and aged neutrophils, 2 key proteins of actin polymerization. These 2 subunits form the core and the branching subunit that stabilize growing actin filaments and are instrumental in migratory processes.^36^ Additionally, JAK2 influences plasma membrane composition by negatively regulating IGF-1 signaling in both young and aged neutrophils.^55,56^ The influence of JAK2 on the release of chemoattractants (decreased SDF-1α in young neutrophils and increased CXCL2 in aged neutrophils) suggests an age-dependent regulation of migration by neutrophils. While young neutrophils appear to respond more robustly to environmental cues and actively suppress chemotactic signals (such as SDF-1α) to guide migration, aged neutrophils may rely more on autocrine signaling. This shift may result in aberrant and dysregulated migration in aged mice due to the use of self-directed cues rather than external/condition-driven cues. Our data suggest that JAK2 has the most direct effect on neutrophil migration; indeed, most of the proteins modulated by pan-JAK inhibition are also modulated by JAK2 inhibition. Overall, these data demonstrate an age-dependent shift in migration regulation.

Very little is known of JAK’s modulation of neutrophil degranulation.^57^ In mast cells, JAK1/2 activity is linked to degranulation in response to inflammatory stimuli.^58^ However, the exact molecular mechanisms are unclear. Our data showed that JAK2 activation regulates degranulation by modulating calcium signaling, SNARE-mediated granule fusion, and actin reorganization. In young neutrophils, JAK2 activation decreased SNARE signaling, leading to the decrease release of primary granules, secondary granules, tertiary granules, and secretory vesicles. In aged neutrophils, JAK2 activation increased the release of secretory vesicles and decreased the release of secondary and tertiary granules. These changes are mediated by JAK2 increase of RAC1/2 (regulator of actin remodeling) activity. These data suggest an age-dependent JAK2 regulation of the release of secretory vesicle content. Emerging evidence suggests that age-related impairments in calcium signaling may contribute to reduced granule release upon activation.^59^ Our data showed that calcium signaling is modulated by JAK2 activity, suggesting an important role of this mechanism in this pathway. Altogether, neutrophil degranulation, unlike secretory vesicle release, is mediated by JAK2 in an age-independent manner.

The role of JAK signaling in NETosis remains unclear, and direct regulation of NET production by JAK2 has not been well established. As previously noted, the only studies examining this have been in myeloproliferative neoplasms, where the JAK2 gain-of-function mutation is associated with increased NET production in human patients.^51,60^ Contrary to this, our findings indicate that JAK2 inhibition alone does not alter NET production under baseline conditions. PAD4, a master regulator of chromatin decondensation and histone citrullination in NETosis, was unchanged across all groups, further supporting the conclusion that JAK2 does not directly drive NET release. However, JAK inhibition did alter NETosis-associated pathways and effector proteins in an age-dependent manner. JAK2 inhibition increased NE in young but not aged neutrophils, left MPO unchanged, and significantly reduced NOX1 in both. Pan-JAK inhibition similarly decreased NOX1 and reduced MPO in aged neutrophils compared to PMA controls. These findings suggest that while JAK2 does not directly regulate NET formation, multiple JAK proteins may modulate early protein events critical for ROS-driven NETosis. These results suggest that JAK proteins influence NETosis indirectly by regulating key mediators, including NOX1, MPO, and NE, and that these effects vary with age.

Neutrophils primarily rely on glycolysis for energy production. A recent study demonstrated that, in neutrophils, glucose can be diverted from glycolysis into PPP to generate NADPH to facilitate ROS production, a process dubbed “glucose shuffling.”^53,61^ Overall, our findings revealed that JAK2 modulates neutrophil metabolism by regulating PPP and glycogen metabolism in an age-dependent manner. In young neutrophils, JAK2 activation increased G6PD, which moves glucose into the PPP cycle.^62^ The activity of G6PD results in the generation of ribulose-5-phosphate, which acts as a substrate for the nonoxidative phase of PPP. Ribose-5-phosphate isomerase, a key enzyme that converts ribulose-5 phosphate to ribose-5-phosphate, a key precursor to the nucleotide synthesis, is upregulated in young neutrophils. TKT facilitates the conversion of ribose-5-phosphate into glyceraldehyde-3-phosphate, a glycolytic intermediate. In young neutrophils, JAK2 signaling increases TKT levels. These data suggest that in young neutrophils, JAK2 activity is important to the generation of NADPH by modulating the movement of glucose into the PPP pathway. In aged neutrophils, JAK2 activation decreases the breakdown of glycogen by reducing AGL levels. AGL breaks down glycogen at α-1,6-glycosidic branch points. This generates linear glycogen branches that PYGL breaks down into glucose molecules.^63^ In the absence of AGL, PYGL activity decreases limiting glucose availability. Our data suggest that aged neutrophils decrease this process with JAK2 activation. In line with previous research showing that glycogen-degrading enzymes decline with age in rat liver,^64^ the aged neutrophils studied here appear to follow a similar trend. This suggests that aging may broadly impair glycogen metabolism across different cell types, potentially limiting the energetic flexibility required for proper neutrophil function. Neutrophils contain a large number of glycogen stores; whether these stores are influenced by age still needs to be determined.^65^ These data suggest that JAK2 regulation of neutrophil glucose metabolism changes with age.

## Conclusion

This study provides new mechanistic insights into JAK2 modulation of neutrophil function. This modulation is influenced by the biological age of the organism. Our data showed that in neutrophils from young and aged mice, JAK2 activation directly modulates migration through membrane composition and cytoskeletal rearrangement. JAK2 activation inhibits release of chemokines in young neutrophils, while increasing its release in aged neutrophils. Our findings indicate that JAK2 activation regulates neutrophil granule release (degranulation) in an age-independent manner, whereas its modulation of secretory vesicle release is age dependent. Finally, JAK2 signaling promotes glucose shuffling to increase PPP activity in neutrophils from young mice, while decreasing glycogen breakdown in neutrophils from aged mice. Together, these data provide evidence that JAK2 modulation of neutrophil function is age dependent.

This study has several limitations that we would like to highlight. Neutrophils in this study were isolated from the bone marrow. As such, we are probing cellular mechanisms in naïve cells. It is established that exiting the bone marrow changes the activation state of neutrophils.^66^ Additionally, the extracted cells likely represent a mix of immature and mature (marginated) neutrophils. Our characterization of the isolates in other studies showed that immature neutrophils make up <1% of the isolated cells (data not shown). This study is focused entirely on mouse neutrophils. Whether these molecular mechanisms are also present in human neutrophils will have to be confirmed in subsequent studies. With mice being the preferred animal to model human disease, we believe it is important to fully understand how these cells are regulated to better interpret future findings. To accurately assess potential off-target effects of the drugs on proteomic changes, mass spectrometry alongside the original samples would have been required; however, this was not feasible, leaving us unable to determine whether the drugs alone induced proteomic alterations. A more definitive strategy would involve evaluating cellular functions in a JAK2 knockout or knockdown system, using either a JAK2^fl/fl^ mouse model or transfection-based approaches. Yet, JAK2^fl/fl^ mice are not commercially available, and current transfection methods would undesirably activate neutrophils, limiting the feasibility of this approach. Despite these constraints, our functional assays demonstrated that key cellular responses were largely preserved and remained comparable to control conditions with baricitinib or AZD1480 administration alone (Fig. S1H, I). We therefore reasoned that any off-target effects were minimal, although further work will be required to fully characterize such effects, particularly in aged mice. Last, to be able to accurately determine which JAK proteins are responsible for these functions and proteomic changes, more experiments would have to be done targeting each JAK protein (JAK1, JAK3, and TYK2). Despite these limitations, this study provides a foundational understanding of how JAK2 activation regulates neutrophil function across biological aging, offering critical insights that may inform future therapeutic strategies targeting immune dysfunction in aging and inflammatory diseases.

## Supplementary Material

Supplemental files

Supplementary material is available at *The Journal of Immunology* online.

## Data Availability

Raw mass spectrometry data underlying this article are available in ProteomeXchange, dataset PXD069013.
