# Supplementary material for: Aging changes the mechanism that underlies JAK2 modulation of neutrophil function
Source: J Immunol. Author manuscript; Available in PMC 2026 Feb 17. (PMC12908130; doi:10.1093/jimmun/vkaf323)
Supplement: Supplementary file 1 [file NIHMS2136105-supplement-Supplemental_files.pdf]

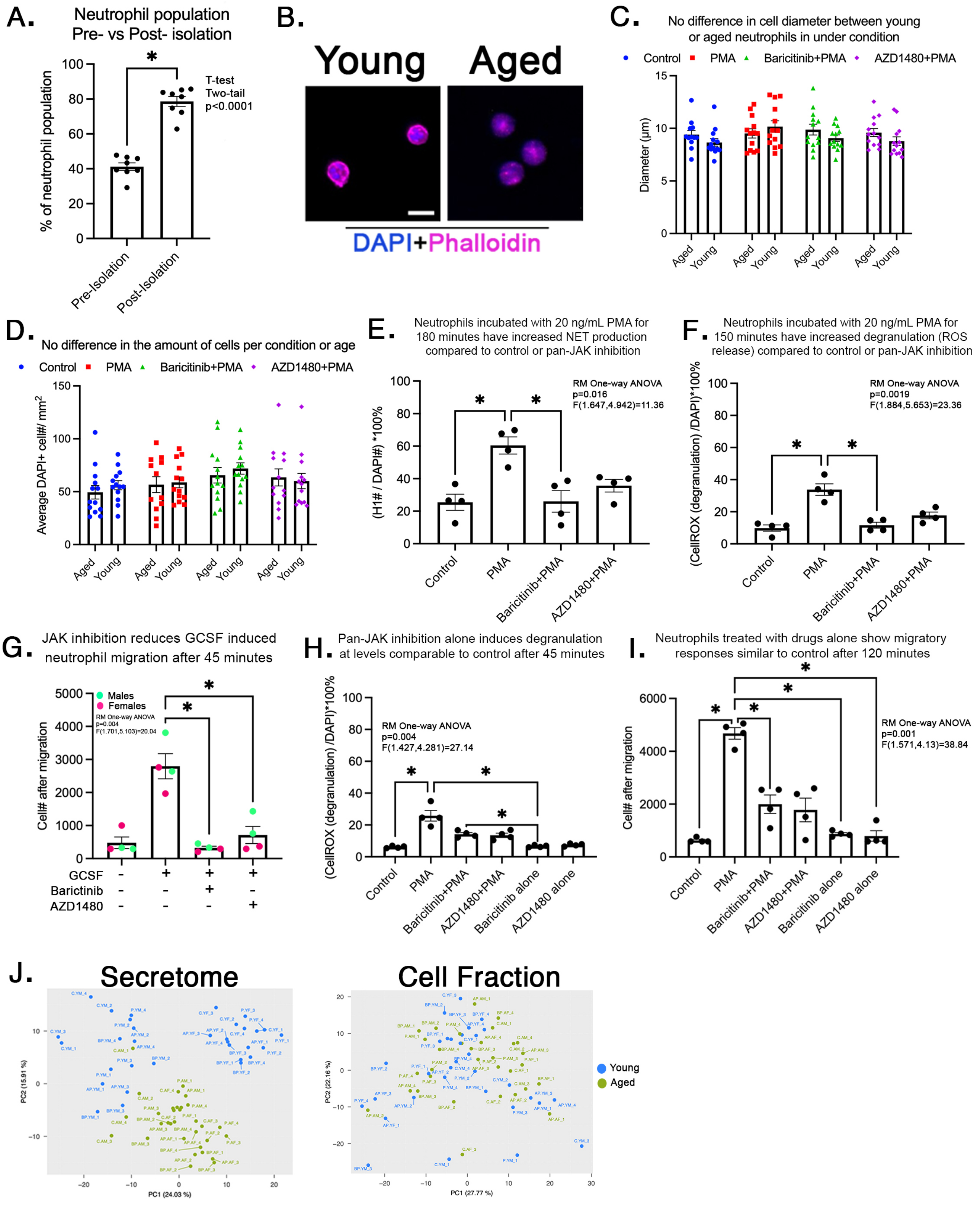

Supplemental Figure 1

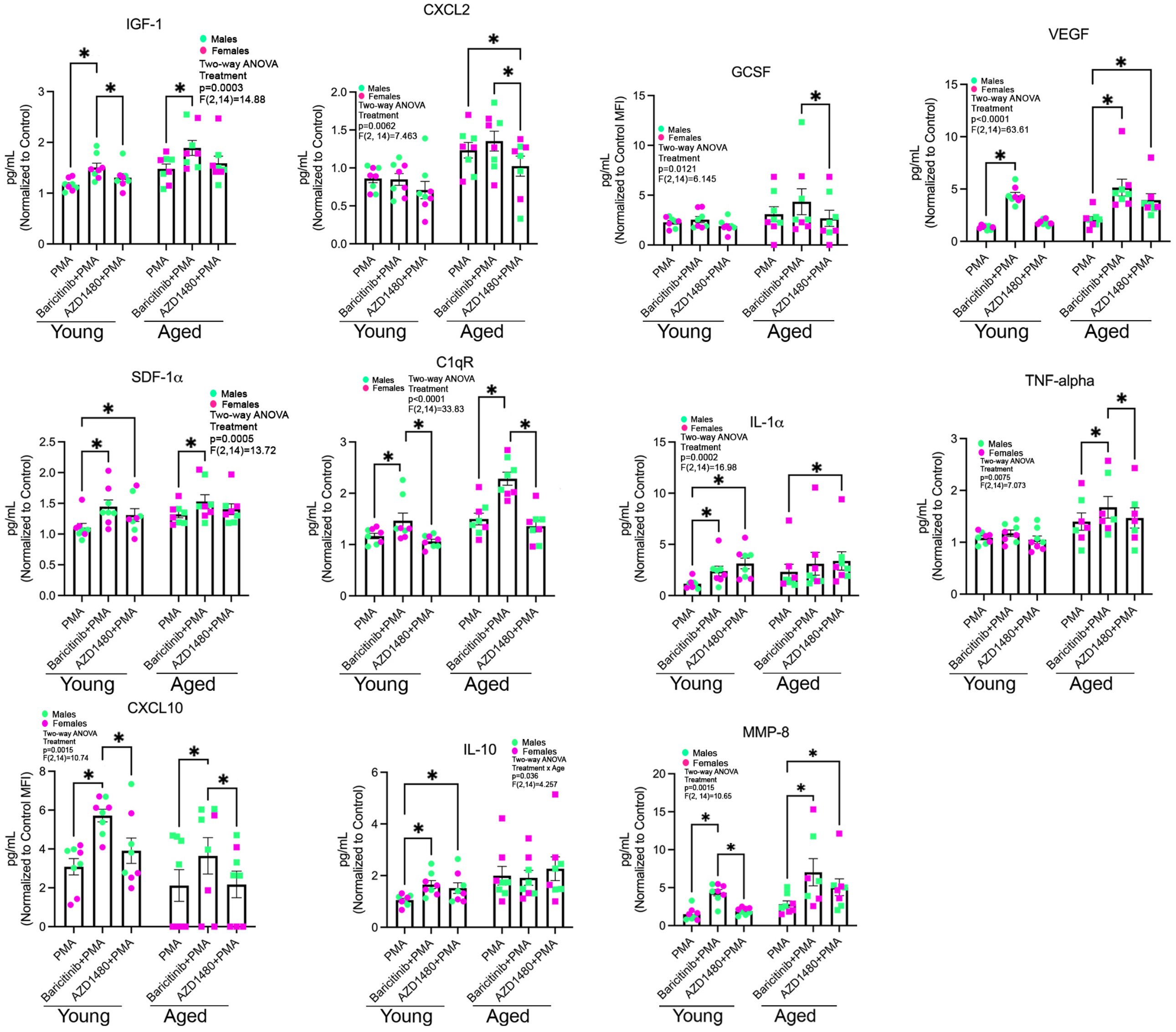

Supplemental Figure 2

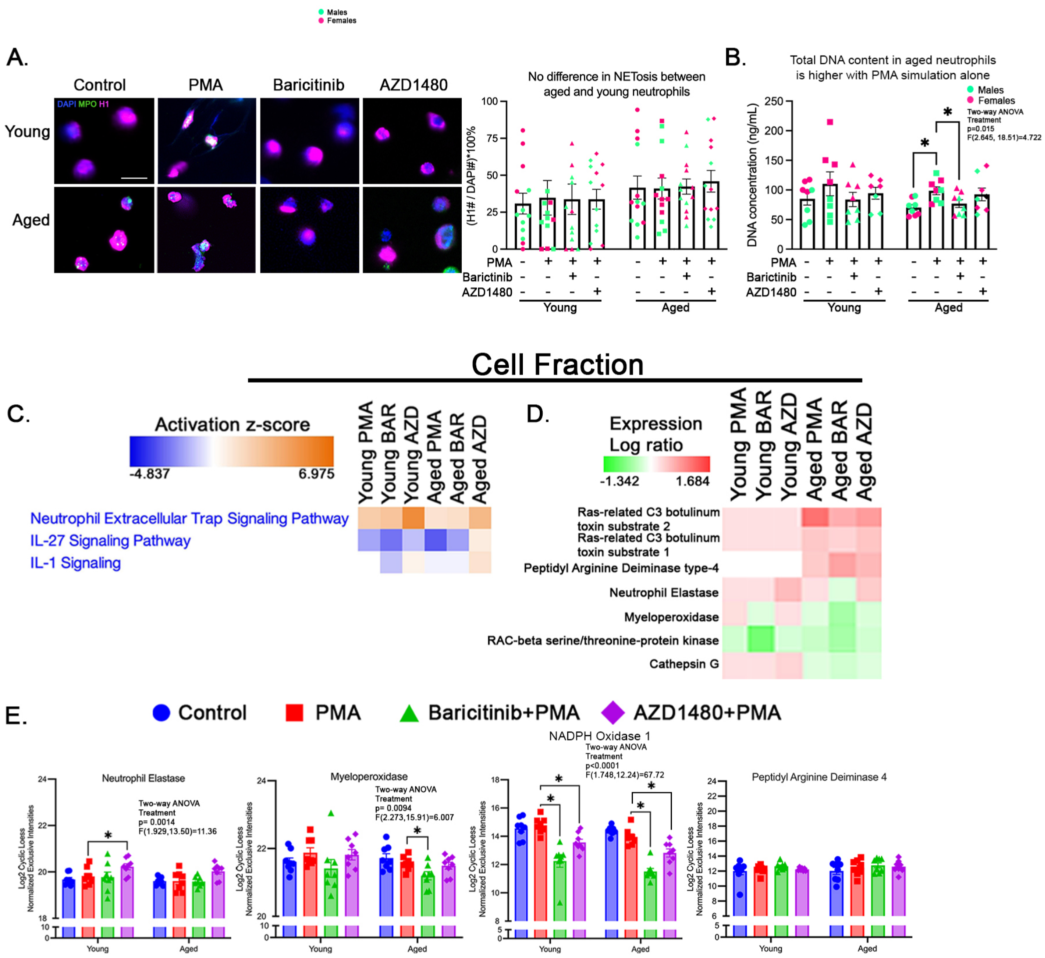

Supplemental Figure 3

Supplemental Figure 1: JAK inhibitors target intended pathway with no change of size, shape and number of neutrophils across conditions. **A.** Quantification of neutrophil population pre- and post-isolation, demonstrating a significant enrichment (~80%) post-isolation (n=8). **B.** Representative fluorescent microscopy images of neutrophils from young and aged mice stained with DAPI (nuclear stain) and phalloidin (actin cytoskeleton) show no difference in morphology, and cell diameter (**C.**) under any conditions. **D.** Cell density (DAPI+ cells per mm^2) is the same across all experimental conditions. Validation of PMA dose showed that PMA increased NET formation at 180 min (**E.**), and degranulation at 150 min (**F.**) in neutrophils from young mice. **G.** G-CSF (1ng/mL) addition increased neutrophil migration after 45 min in the absence of PMA, in neutrophils from young mice. This increase was abolished in the presence of Baricitinib and AZD1480. Assessment of drug off target effect showed that degranulation (**H.**) and migration (**I.**) were not affected by Baricitinib nor AZD1480 in the absence of PMA, in neutrophils from young mice. **J.** PCA plots of mass spectrometry data showed a separation based on sex in neutrophils from young mice and no separation in neutrophils from aged mice. No separations were observed between groups in the cell fraction remains. p-value: * <0.05; bar graphs represent mean ± SEM. (A-I) Individual points corresponding to individual mice (pink= females, green= males).

Supplemental Figure 2: Neutrophils from young and aged mice exhibit distinct cytokine, chemokine, and growth factor secretion profiles in response to JAK inhibition. Supernatants from neutrophils from young and aged mice bone marrow-derived neutrophils were collected and activated with PMA for 45 minutes, with or without pre-treatment for 1 hour with either pan-JAK inhibitor (Baricitinib) or JAK2-selective inhibitor (AZD1480). Luminex multiplex assay was performed to quantify secretion of multiple cytokines (IL-1α, IL-10, TNF-α), chemokines (CXCL2, SDF-1α, CXCL10), growth factors (IGF-1, VEGF, GCSF), matrix metalloproteinase (MMP-8), and complement receptor (C1qR). Panels display normalized levels (pg/mL or MFI, as indicated). *p<0.05; bar graphs represent mean ± SEM. Individual points corresponding to individual mice (pink= females, green= males).

Supplemental Figure 3: JAK activity regulates NADPH oxidase 1 with little effect on NETosis. **A.** Representative images of NET (DAPI+histone1+MPO+) in neutrophils from young and aged mice under all conditions. Quantification of images shows that no condition effect on NET production. **B.** Assessment of free DNA, using PicoGreen assay, showed that at 45 min post incubation only neutrophils from aged mice had increased DNA release, which was decreased in the presence of Baricitinib. **C.** Functional heatmap of cell fraction showed the NET signaling pathway did not change in the neutrophils from young or aged mice with AZD1480 nor Baricitinib treatment compared to PMA. AZD1480 treated neutrophils from aged mice had increased IL-27 and IL-1 signaling compared to PMA. Baricitinib treated neutrophils from young mice decreased IL-1 signaling. **D.** Heatmap illustrating cell fraction protein abundance (log ratio) changes in NETosis-related proteins. AZD1480 treated neutrophils from young mice had no change in protein abundance of listed proteins, except an increase in neutrophils elastase (NE) and cathepsin G compared to PMA. Neutrophils from aged mice treated with AZD1480 showed no change in the listed proteins compared to PMA. Baricitinib treated neutrophils from young mice had no change in proteins listed, except for a decrease in MPO and RAC-beta serine/threonine-protein kinase compared to PMA. Neutrophils from aged mice treated with Baricitinib showed no change in all listed proteins compared to PMA. **E.** Bar graphs show the that the relative expression of NE is increased in neutrophils from young mice with AZD1480

treatment compared to PMA. MPO is significantly decreased with Baricitinib treatment compared to PMA treatment in neutrophils from aged mice. In both neutrophils from young and aged mice, NOX1 is significantly decreased with Baricitinib and AZD1480 treatment compared to PMA. PAD4 remains unchanged with all conditions. Scale bar= 20μm; bar graphs represent mean ± SEM; p-value: * <0.05; (A-B and E) Individual points corresponding to individual mice (pink= females, green= males).
